# A novel NAC transcription factor mediates negative regulation of early ethylene production and ripening in tomato fruits

**DOI:** 10.3389/fpls.2025.1696915

**Published:** 2025-11-19

**Authors:** Jiawei Li, Junwen Han, Tong Li, Daoyun Chen, Kenji Miura, Lazaro Eustaquio Pereira Peres, Hiroshi Ezura, Ning Wang

**Affiliations:** 1 Graduate School of Life and Earth Sciences, University of Tsukuba, Tsukuba, Japan; 2 Institute of Life and Environmental Sciences, University of Tsukuba, Tsukuba, Japan; 3 Tsukuba Plant Innovation Research Center, University of Tsukuba, Tsukuba, Japan; 4 Department of Biological Sciences, College of Agriculture ‘Luiz de Queiroz’ (ESALQ), University of São Paulo, Piracicaba, São Paulo, Brazil

**Keywords:** fruit ripening, postharvest physiology, ethylene biosynthesis regulation, ripening onset control, light intensity response, NAC transcription factor

## Abstract

Timely initiation of fruit ripening is crucial for improving agricultural efficiency and shelf life. While the progression of tomato ripening and the roles of ethylene and its core transcriptional controls are well established from the breaker (BR) stage onwards, the molecular mechanisms that fine-tune the transition from fruit development to ripening remain poorly understood. In this study, we identified a previously uncharacterized NAC transcription factor (TF), *Ripening Accelerator* (*RAR*), as a key negative modulator of climacteric ripening onset. In fruit, *RAR* is highly expressed at the mature green (MG) stage and downregulated at BR stage, preceding the climacteric ethylene burst. Silencing *RAR* via RNA interference significantly accelerated fruit ripening and ethylene production prior to BR stage, especially under high light conditions. RAR directly represses *ACC Synthase 2* (*ACS2*), a key ethylene biosynthesis gene. Although RAR can form a heterodimer with the ripening-promoting NAC TF Non-Ripening (NOR), this heterodimer exhibits weaker transcriptional activation than the NOR homodimer, indicating a repressive effect of *RAR* on *NOR*-mediated activation. Moreover, *RAR* expression is negatively regulated by ethylene, forming a feedback loop that modulates the timing of ripening onset. Our findings uncover a previously unrecognized regulatory checkpoint in the ripening program, where *RAR* probably acts as a developmental safeguard to prevent premature ripening. Targeted manipulation of *RAR* offers a promising strategy for fine-tuning ripening onset and improve postharvest fruit quality across diverse environmental conditions.

## 1 Introduction

The control of fruit maturation is a valuable trait that continues to be selected for breeding programs to improve agricultural efficiency. For instance, fruits that mature more quickly consume less water, fertilizer, and energy over their lifespan, increase crop productivity, and contribute to more sustainable agriculture (Morcillo et al., 2021). Faster maturing varieties can avoid peak supply periods and gain a price advantage by capturing the market ahead of time (Garg et al., 2024). On the other hand, in some cases, delaying fruit maturation may be advantageous when the goal is to improve fruit nutrient content and quality (Ju et al., 1999). However, despite substantial progress in understanding fruit ripening, there is still limited information regarding the molecular mechanisms that regulate the transition from reproductive development to ripening.

Fruit development progresses through three distinct phases: i) Growth Phase, which begins immediately after fertilization and is characterized by rapid cell division and expansion; ii) Maturation Phase, during which the fruit attains its full size and seed development is completed; and iii) Ripening Phase, in which physiological and biochemical changes occur, including modifications in color, texture, and flavor, making the fruit suitable for consumption and facilitating seed dispersal (Gillaspy et al., 1993; Giovannoni, 2004). These modifications also result in the rich flavor and aroma of the ripe fruit (Gray et al., 1994). Based on respiratory changes during ripening, fruits are categorized into two groups: climacteric and non-climacteric groups. The ripening of non-climacteric fruits is primarily regulated by abscisic acid (ABA) (Fenn and Giovannoni, 2021), while the ripening of climacteric fruits is thought to be mainly controlled by ethylene (Millerd et al., 1953). In the Solanaceae family, besides the climacteric tomato, there are non-climacteric fruits like eggplant and pepper (Moore et al., 2005). Some wild tomato species, such as S. *pennellii* and S. *peruvianum*, also display non-climacteric characteristics (Grumet et al., 1981).

In climacteric fruits, such as tomatoes, the onset of the ripening process is marked by increased ethylene production, which induces changes in color, aroma, texture, and flavor (Lelièvre et al., 1997). Ethylene biosynthesis involves the amino acid methionine, which is converted to S-adenosylmethionine (SAM), while ACC synthase (ACS) catalyzes the reaction to form 1-aminocyclopropane-1-carboxylic acid (ACC). Finally, the oxidation step by ACC oxidase (ACO) leads to the formation of ethylene (Adams and Yang, 1979). Ethylene is involved in various physiological processes in higher plants, including fruit ripening and leaf senescence (Iqbal et al., 2017). Ethylene production in climacteric fruits comprises two systems: the autoinhibitory System 1, functioning in immature fruits, and the autocatalytic System 2, which dominates during ripening (Lelièvre et al., 1997). In tomatoes, ethylene production is mainly regulated by four ACS isozymes (ACS1A, ACS2, ACS4, ACS6) and three ACO isozymes (ACO1, ACO3, ACO4), each of which is associated with either Systems 1 or System 2 ethylene production (Paul et al., 2012). Besides promoting ripening, ethylene influences the expression of genes involved in its own biosynthesis pathway via different TFs. The MADS-box TF *Ripening-Inhibitor* (*MADS-RIN*) was the first identified regulator of *ACS* that directly enhances its expression during fruit ripening (Robinson and Tomes, 1968). In addition to MADS-box TFs, the NAC (NAM, ATAF1/2, and CUC2) TF family, unique to plants, is one of the most prominent TF families involved in various developmental processes, including fruit ripening (Forlani et al., 2021). *Non-Ripening* (*NAC-NOR*) is a key regulator of fruit ripening initiation and progression (Giovannoni, 2004, Giovannoni, 2007). The roles of ethylene, *MADS-RIN*, and *NAC-NOR* in the ripening regulatory network are well established. *MADS-RIN* directly regulates *ACS2*, *ACS4* (Fujisawa and Yasuhiro, 2013) and *ACO1* (Lü et al., 2018). *NAC-NOR* also contributes to ripening by activating the expression of *ACS2* and *ACO3* (Gao et al., 2020; (Wang et al., 2018). Ethylene, in turn, induces the expression of *MADS-RIN* and *NAC-NOR*, reinforcing the autocatalytic loop that drives ripening (Giovannoni et al., 2017). Other NAC TFs, including *Non-Ripening like1* (*SlNOR-like1*) (Gao et al., 2018), *SlNAC1* (Meng et al., 2016), *SlNAC4* (Zhu et al., 2014) have also been implicated in ripening, with additional members likely to be identified.

The fruit Maturation Phase in tomato is a consequence of the action of ethylene, the main plant hormone also responsible for coordinating transcriptional activation of genes associated with the Ripening Phase in tomato. Ethylene biosynthesis transition from System 1 to System 2 is developmentally regulated, involving delicately orchestrated changes in gene expression levels of *ACS* and *ACO*. Most known regulators, such as *RIN* and *NOR*, function during the BR stage, predominantly influencing System 2 ethylene production. In contrast, the regulatory events occurring during the transition from System 1 to System 2, when the fruit begin ripening, remain poorly understood despite their potential to influence ripening initiation without compromising fruit development. Notably, the MG stage marks the end of reproductive growth. Inducing earlier ripening around the BR stage, when the fruit is already physiologically competent, could accelerate ripening while keeping the adverse effects on fruit quality to a minimum. The Tomato Expression Atlas provides high-resolution gene expression profiles during fruit development and ripening, which is a significant resource to identify candidate genes with increased expression prior to the BR stage and correlated with ethylene biosynthetic genes (Fernandez-Pozo et al., 2017). In this study, transcriptional profiling at the onset of ripening revealed a subset of previously uncharacterized NAC TFs whose expression patterns closely parallel those of *ACS* and *ACO* family genes. Among them, we focused on a novel NAC family gene, hereafter referred to as *Ripening Accelerator* (*RAR*), whose expression precedes the BR stage and is likely regulated by developmental cues. Our data provided insights into the role of NAC TFs in regulating the transition from System 1 to System 2 ethylene production, highlighting their potential as fine-tuning regulators of fruit ripening onset. These findings may provide a new strategy to improve postharvest quality and extended shelf life through early-stage genetic modulation.

## 2 Materials and methods

### 2.1 Plant materials and growth conditions

Two different ‘Micro-Tom’ (*Solanum lycopersicum* L.) wild-type backgrounds (WT-J and WT-B) were used in this study, originating from Japan and Brazil, respectively. The wild-type WT-J was obtained from the National Bioresource Project tomato (NBRP tomato: http://tomato.nbrp.jp/indexEn.html). The *nor*, *rin*, and WT-B lines used for qRT-PCR analysis were provided by the University of São Paulo, Brazil, where their molecular and phenotypic characteristics were analyzed. Plants were grown from October 2023 to January 2024 in the greenhouse at the Tsukuba Plant Innovation Research Center (T-PIRC), University of Tsukuba. The plants were maintained under 16 hours of light (82 µmol photons m^-^² s^-^¹ as normal light intensity, 235 µmol photons m^-^² s^-^¹ as high light intensity)/8 hours dark photoperiod at a constant temperature of 24°C. NPK supplementation was performed once per week using a 500-fold diluted HYPONeX solution (Hyponex, Osaka, Japan). The date of pollination was recorded for each flower to track fruit development. Upon harvest, seeds and columella tissues were immediately excised and rapidly frozen in liquid nitrogen and stored at -80°C for subsequent experiments.

### 2.2 Target gene selection

Tomato gene expression data were obtained from the Tomato Expression Atlas (Cornell University, http://tea.solgenomics.net/). As the first criterion for comparison, the maximum averaged RPM (Reads Per Million) among all samples was used, which represents the expression level at the time point with the highest average expression across different stages and tissues of tomato fruit ripening. Additionally, the expression profiles of all NAC transcription factor family members were visualized and analyzed using Morpheus (Broad Institute, https://software.broadinstitute.org/morpheus/). As a second criterion, gene expression patterns were evaluated based on the trend of expression level changes corresponding to distinct fruit ripening stages. The unpublished gene with the highest overall expression in fruit among the NAC family was selected as a candidate gene for regulating fruit ripening and subjected to functional validation.

### 2.3 Development of transgenic tomato lines

Gene-specific primers (**Supplementary Table 2**) were designed and utilized to amplify the sequence *RAR* for RNA interference (RNAi) vector construction. The amplified fragment of *RAR* was purified by QIAquick PCR Purification Kit (QIAGEN, Hilden, Germany) and cloned into the entry vector pCR™8/GW/TOPO™ using the TA Cloning Kit (Invitrogen, Waltham, MA, USA). The vector was subsequently transformed into competent cell of *Escherichia coli* strain DH5α. Positive clones with correct sequence were identified, and plasmids were extracted and recombined with the destination vector (pBI_sense, antisense_GW) by LR reaction (Gateway™ LR Clonase™ II Enzyme mix, Invitrogen, Waltham, MA, USA).

The full-length coding sequence of *RAR* was amplified from cDNA using gene-specific primers (**Supplementary Table 2**) and KOD-Plus-Neo polymerase (TOYOBO, Osaka, Japan) for overexpression vector construct. The purified PCR product was inserted into the binary vector pBI121 using the In-Fusion^®^ HD Cloning Kit (Takara Bio, Shiga, Japan) following double digestion with *Sac*I and *Sma*I. The resulting constructs were transformed into *Escherichia coli* strain DH5α, and positive clones were selected on LB agar plates containing kanamycin. Positive clones with correct sequence were identified and plasmids were extracted using the QIAprep Spin Miniprep Kit (QIAGEN, Hilden, Germany).

The resulting recombinant plasmid for RNAi and overexpression was introduced into *Agrobacterium tumefaciens* strain GV3101 by electroporation. For plant transformation, Micro-Tom WT-J seeds were surface-sterilized, germinated on Murashige and Skoog (MS) medium, and the 5-day-old cotyledons were excised for Agrobacterium-mediated infection. Each cotyledon was bisected into 2 equal halves, and the cut surface was thoroughly immersed in the Agrobacterium infection solution. After a 30-minute incubation period, excess infection solution was removed with sterile Kimwipes. The cotyledon halves were then placed cut surface down onto the co-culture medium (**Supplementary Table 1**). The plates were subsequently wrapped in aluminum foil and placed in an incubator maintained at 25°C. After 2 days of cultivation, bacterial colonization around the cut sections was observed, and the sections were transferred to callus culture medium (**Supplementary Table 1**). The callus formation was observed within 14 to 21 days. The Calli were then transferred to shoot induction medium (**Supplementary Table 1**) for 20 days of cultivation, and regenerated shoots were excised and transferred to rooting induction medium for 14 days of cultivation (**Supplementary Table 1**). Transgenic plants were screened via PCR using NPTII gene-specific primers (**Supplementary Table 2**) and subsequently transplanted into sterile soil for acclimatization. To confirm the ploidy status of the transgenic plants, plant leaves were cut into 5 mm squares and placed in a petri dish. For nuclear staining of plant cells, 150 μL of DAPI staining solution from the Plant Ploidy Analysis DNA Reagent Kit (Sysmex Partec GmbH, Goerlitz, Germany) was applied. The sample was then chopped with a razor blade to extract the nuclei. The sample liquid was filtered through a mesh and transferred into a plastic test tube, containing 600 μL of nuclei extraction buffer from the above-mentioned kit. The DAPI fluorescence intensity was subsequently measured using a flow cytometer. Diploid plants were retained.

### 2.4 Plant growth analysis

We measured 4 plants each genotype for plant growth. The plant height under normal light and high light were measured between cotyledon and apical bud at DAS (Days after sowing) 58 and 33, respectively. Chlorophyll content was measured by clamping three random points on each leaf with SPAD-502Plus analyzer (Konica Minolta, Tokyo, Japan). A total of 6 leaves were randomly selected between the second and third inflorescence of both WT-J and RNAi-RAR 17–8 T_2_ plants at DAS 61.

### 2.5 Ethylene production measurement

Ethylene production was measured in the fruits of WT-J and homozygous RNAi-RAR 17–8 T_2_ plants at 18, 23, 26, 29, 32, and 36 days after pollination. The fruits were carefully placed in 50 mL vial bottles to prevent mechanical damage. To eliminate the effect of wound-induced ethylene production, the bottles were left open for 1 hour after fruit placement, allowing for air exchange. Subsequently, the air inside the bottles was replaced by gentle airflow to further remove the residual wound-induced ethylene. Following this pretreatment, the bottles were tightly sealed and incubated at room temperature for 2 hours. A 1mL sample of headspace gas was collected using a medical syringe and injected into a SGC (Sensor Gas Chromatograph) SGEA-P3-C1/C2/C3 (NISSHA, Osaka, Japan) equipped with a flame ionization detector (FID) for ethylene quantification. The ethylene concentration was normalized to reagent-grade ethylene standards and expressed relative to fruit weight, bottle volume, and incubation time using the following equation:


$$\text{Ethylene concentration}(\text{μL}/\text{g fresh weight}/\text{h})=\frac{X\times Y}{Z\times T}$$

Where:

- X is the concentration (ppm),
- Y is the volume of the bottle (mL),
- Z is the fruit weight (g),
- T is the time (h).

Each measurement was performed using three biological replicates for both wild-type and transgenic fruit samples.

### 2.6 Exogenous ethylene treatment of fruits

The Micro-Tom WT-J and RNAi-RAR 17–8 fruits at BR stage were harvested and placed in 50 mL open vial bottles for 1 hour to eliminate the effect of wound-induced ethylene production. Subsequently, the fruits were exposed to an exogenous ethylene treatment by incubating them in a sealed chamber containing 1000 ppm of ethylene at room temperature for 24 hours. Fruits subjected to ambient air treatment under identical conditions were used as controls. Following the treatment, the fruit pericarp of the fruits was quickly frozen in liquid nitrogen and stored at -80°C for subsequent experiments. Each treatment was conducted with three biological replicates, and each replicate comprised five individual tomato fruits.

### 2.7 RNA extraction and quantitative real-time PCR

Total RNA was extracted from 100 mg of frozen fruit tissue powder using TRIzol (Invitrogen, Waltham, MA, USA) following the manufacturer’s instructions. At least three biological replications were included for each sample. The quality of total RNA was assessed by agarose gel electrophoresis using 2×RNA loading buffer (FUJIFILM Wako, Osaka, Japan). The concentrations of total RNA were measured using a Multiskan spectrophotometer (Thermo Fisher Scientific, Waltham, MA, USA). Complementary DNA (cDNA) was synthesized from total RNA using ReverTra Ace qPCR RT Master Mix. subsequently the cDNA was 5 times diluted and used as the template for qRT-PCR. The qRT-PCR analysis was performed using a StepOne Plus real-time PCR system (Applied Biosystems, Foster City, CA, USA). Each reaction was conducted in a 10 μL reaction volume, containing 5 μL of THUNDERBIRD Next SYBR qPCR Mix (Toyobo, Osaka, Japan), 10 nM of forward and reverse primers (**Supplementary Table 3**), 2 μL of cDNA template, and 2.4 μL of MilliQ water. The amplification program was set as follows: 95°C for 20 s, followed by 40 cycles of 95°C for 10 s and 60°C for 30 s. Gene expression levels were quantified using the 2^−ΔΔCt^ analysis method, with Ubiquitin serving as the internal reference gene for normalization.

### 2.8 Biomolecular fluorescence complementation assay

For the BiFC assay, the coding DNA sequences (CDS) of RAR and NOR were amplified by PCR using gene specific primers (**Supplementary Table 2**). The resulting PCR products were cloned into the pRI201-cEYFP-C1 and pRI201-nEYFP-C1 vectors, respectively, to generate the constructs pRI201-cEYFP-RAR-C1 and pRI201-nEYFP-NOR-C1 for further analysis. Additionally, the full-length cDNA of AHL22 (Yun et al., 2012) was amplified by PCR and inserted into the pEAQ-mCherry vector to generate the pEAQ-AHL22-mCherry construct, which served as a nuclear localization control. The three constructs were mixed in equal ratios (1:1:1) and co-infiltrated into leaves of 4-week-old tobacco (*Nicotiana benthamiana*) plants using the method described by Sparkes et al. (2006). Following incubation at 24°C for 24–48 hours, YFP fluorescence signals were observed using a Zeiss LSM700 laser scanning confocal microscope (Carl Zeiss, Jena, Germany).

### 2.9 Dual-luciferase reporter system assay

For the dual-luciferase reporter assay, the upstream promoter sequence of *ACS2* (1 kb) and *ACO1* (922 bp) was cloned and inserted into pGreen II 0800-LUC, and the CDS of *RAR* and *NOR* were cloned and inserted into a pBI121 vector, respectively. The primers are listed in **Supplementary Table 2**. Then, the pGreen II 0800-LUC-*ACS2*, pGreen II 0800-LUC-*ACO1* were transformed into *Agrobacterium tumefaciens* strain GV3101 with the pSoup helper plasmid, and the pBI121-*RAR* was transformed into *Agrobacterium tumefaciens* strain GV3101. Agrobacterium containing pGreen II 0800-LUC-*ACS2*/*ACO1* and pBI121-*RAR* was expanded to OD_600_ = 0.5 in liquid LB medium, respectively. The method for the dual-luciferase reporter system assay was carried out as described by (Yoo et al., 2007). The ratio of transactivation activities of firefly luciferase and renilla luciferase was determined using the Dual-Luciferase^®^ Reporter (DLR™) Assay System according to the manufacturer’s instructions (E1910; Promega, Madison, WI, USA).

### 2.10 Statistical analysis

**Figure 1:**
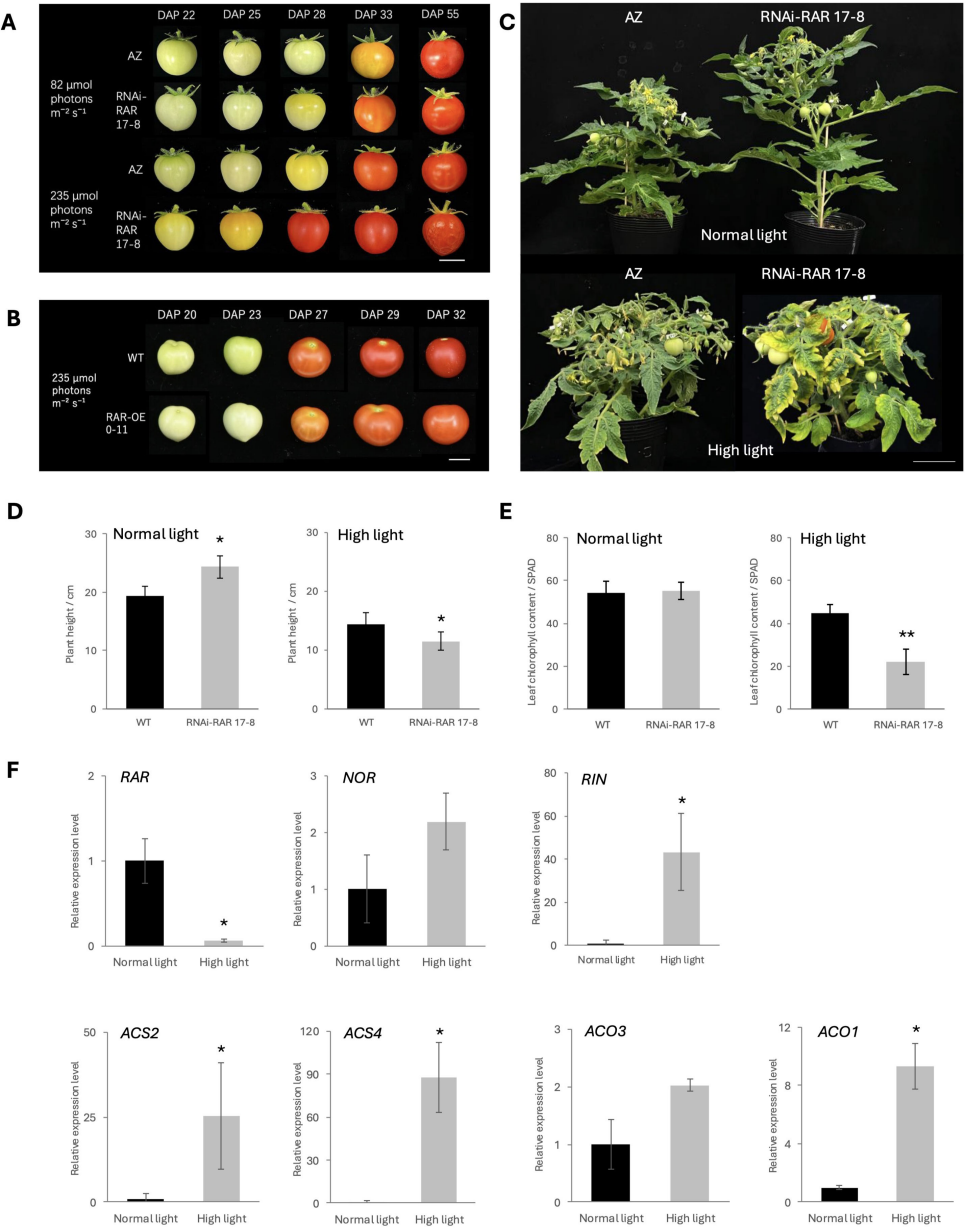
Accelerated fruit ripening in RNAi-RAR 17-8. **(A)** Fruit phenotype of AZ (azygous) and RNAi-RAR 17–8 exposed to controlled light conditions of 82 µmol photons m^-^² s^-^¹ and 235 µmol photons m^-^² s^-^¹, respectively. Bar = 1 cm. **(B)** Fruit phenotype of WT and RAR-OE 0–11 exposed to controlled light conditions of 235 µmol photons m^-^² s^-^¹. Bar = 1 cm. **(C)** Plant growth status of AZ and RNAi-RAR 17–8 plant at DAS (Days after sowing) 58 and 61, respectively. Bar = 10 cm. **(D)** Plant height of WT-J and RNAi-RAR 17–8 plant at DAS 58 and DAS 33, respectively. Error bars represent the standard deviations. Significant differences were determined by two-tailed Student’s t test (*p < 0.05), n = 4. **(E)** Chlorophyll content of WT-J and RNAi-RAR 17–8 leaves at DAS 61. Error bars represent the standard deviations. Significant differences were determined by two-tailed Student’s t test (**p < 0.01), n = 6. **(F)** Relative expression levels of ethylene biosynthesis genes (*ACS2*, *ACS4*, *ACO1*, *ACO3*) and transcription factors (*RAR*, *NOR*, *RIN*) in MG stage fruits of WT grown under normal light and high light (82 µmol photons m^-^² s^-^¹, 235 µmol photons m^-^² s^-^¹). Bars represent mean ± SD (n = 3 biological replicates). Different letters indicate significant differences according to Tukey’s test (p < 0.05).

**Figure 2:**
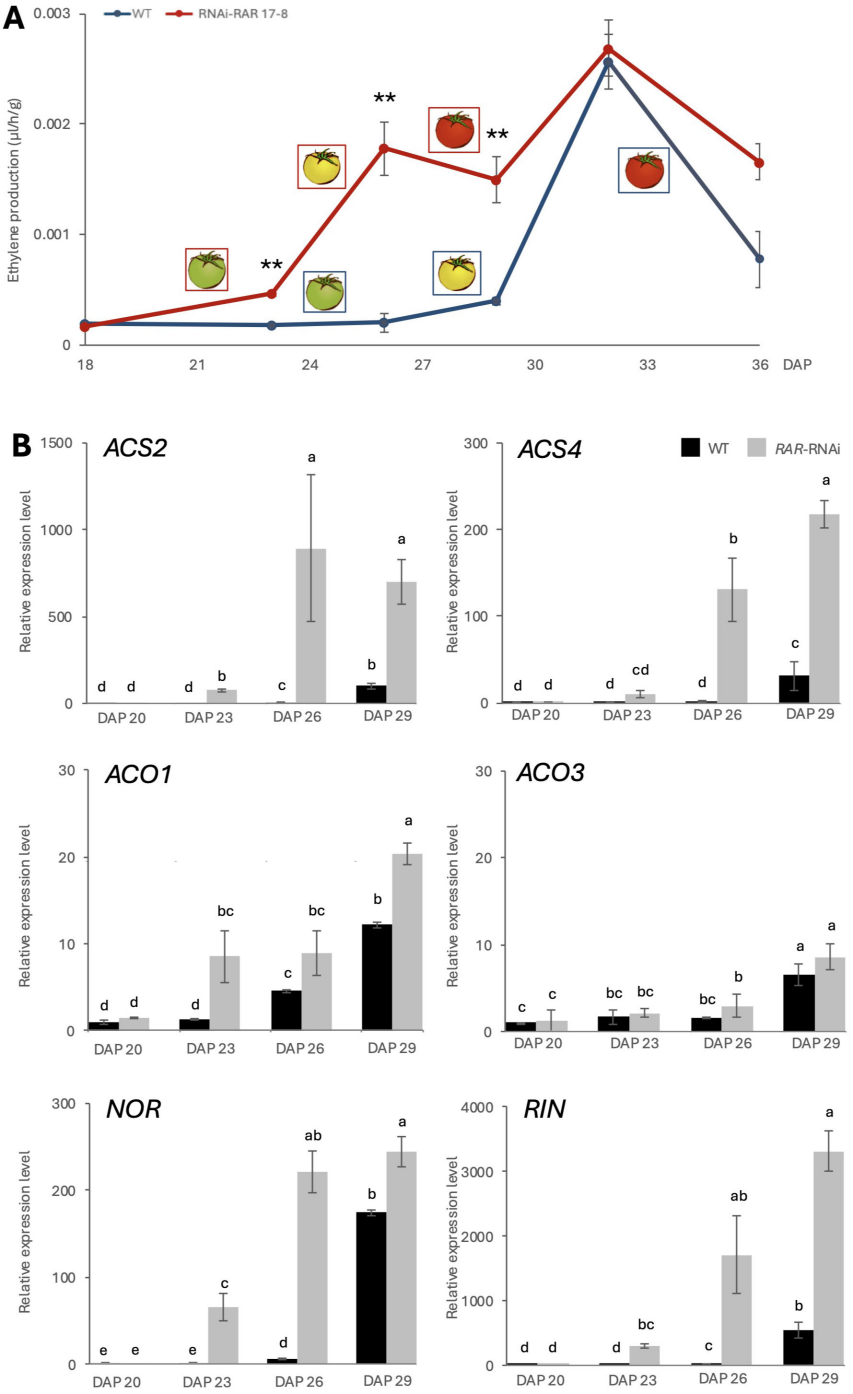
Ethylene production and related gene expression. **(A)** Gas chromatography (GC) analysis of ethylene production from WT-J and RNAi-RAR 17–8 fruits. Error bars represent the standard deviations. Significant differences were determined by two-tailed Student’s t test (**p < 0.01), n = 5. **(B)** Relative expression level of ethylene biosynthesis genes (*ACS2*, *ACS4*, *ACO1*, *ACO3*) and key fruit ripening transcription factors (*NOR*, *RIN*) in RNAi-RAR 17–8 lines compared to WT-J from DAP23 to DAP29. Error bars represent the standard deviations. Letters indicate significant differences according to Tukey’s test (p < 0.05), n = 3. DAP means “Days After Pollination”.

**Figure 3:**
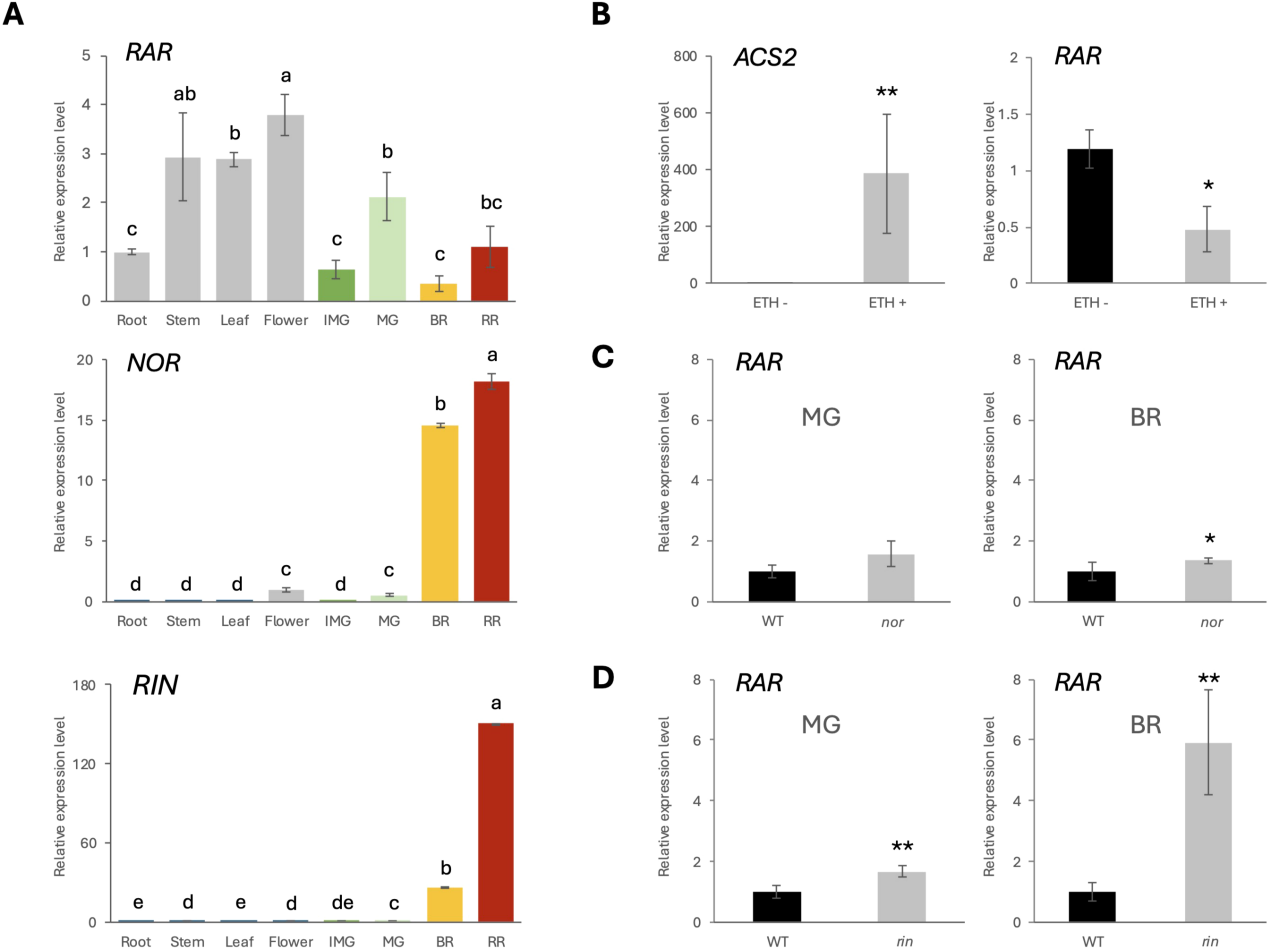
Interregulation between RAR, NOR and RIN. **(A)** Relative expression level of *RAR* in WT-J root, stem, leaf, flower and fruits at IMG, MG, BR, RR stage. Error bars represent the standard deviations. Letters indicate significant differences according to Tukey’s test (p < 0.05), n = 3. **(B)** Relative expression level of *ACS2* and *RAR* in WT-J MG fruits with exogenous ethylene treatment (1000 ppm for 24 hours). **(C, D)** Relative expression level of *RAR* in WT-B, *nor* and *rin* mutants at MG or BR stage, respectively. Error bars represent the standard deviations. Significant differences were determined by two-tailed Student’s t test (*p < 0.05; **p < 0.01), n = 3.

**Figure 4:**
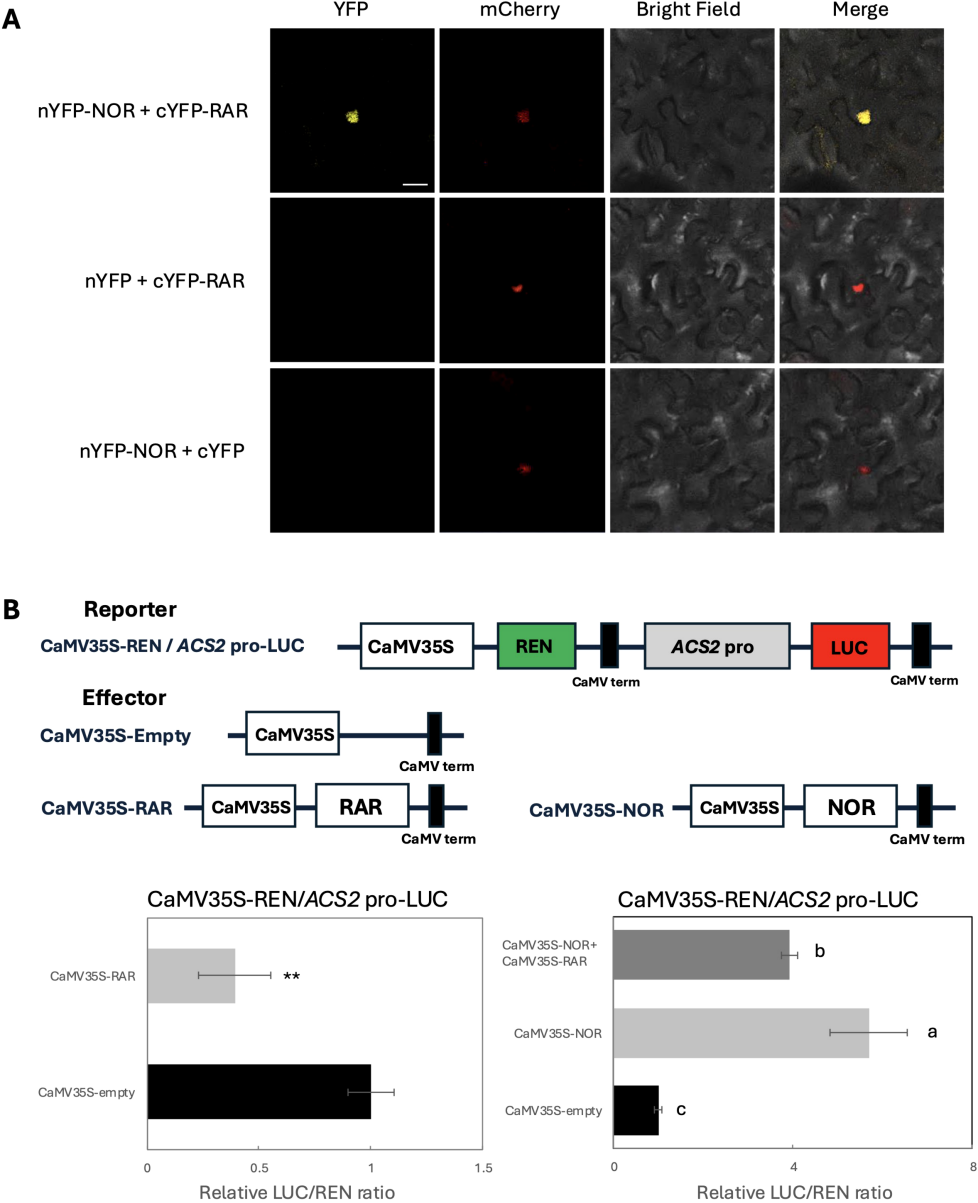
Down regulation of NOR-mediated activation of the *ACS2* promoter through RAR-NOR interaction. **(A)** BiFC assays showing the molecular interaction between RAR and NOR. *AHL22*-mCherry was used as a nuclear marker. Scale bars: 20 µm. **(B)** Dual-luciferase assays showing the effect of RAR on the transcriptional activity of *ACS2*. *RAR* and *NOR* were driven by 35S promoter as an effector. 1kb of *ACS2* promoter were used to drive LUC as a reporter. The activation of *ACS2* promoter by RAR was shown by the ratio of LUC to REN. Data are the mean ± SD of 5 biological replicates. Letters above the bars of the figure on the left indicate statistically significant differences (Student’s t test; **p < 0.01), n = 5. Error bars of figure on the right represent the standard deviations. Letters indicate significant differences according to Tukey’s test (p < 0.05), n = 5.

All statistical analyses and data processing were performed using Microsoft Excel (version 16.95.1). Data was presented as mean± standard deviation. Significant differences in **Figures 1C–F**, **2A**, **3B–D**, **4B**, and **Supplementary Figures 2**–**4** were analyzed using two tailed Student’s t-test (*p < 0.05, **p < 0.01). Multiple comparison was performed via Tukey’s test in **Figures 2B**, **3A** and **4B**, with statistical significance defined as p < 0.05.

## 3 Results

### 3.1 Differential expression of *RAR* and NAC family genes during fruit development

Gene expression data of the NAC TF family in fruits were retrieved from the Tomato Expression Atlas (Cornell University, http://tea.solgenomics.net). NAC genes exhibiting a maximum averaged Reads Per Million (RPM) value greater than 5 were considered for further analysis. The two most highly expressed genes were *NOR* (*Solyc10g006880*, with a max averaged RPM = 916.996) and *SlNAC4* (*Solyc11g017470*, with a max averaged RPM = 742.87). Both NAC family genes have been previously characterized and functionally validated in the context of fruit ripening. Accordingly, the gene displaying the third highest expression level, *Solyc03g080090*, was selected as a novel candidate NAC TF potentially involved in the regulation of ripening (**Supplementary Figure 1**). As subsequent studies showed that knockdown of this gene accelerates fruit ripening, we named the RNAi knockdown mutant of *Solyc03g080090* as *Ripening Accelerator* (*RAR*).

**Figure 5:**
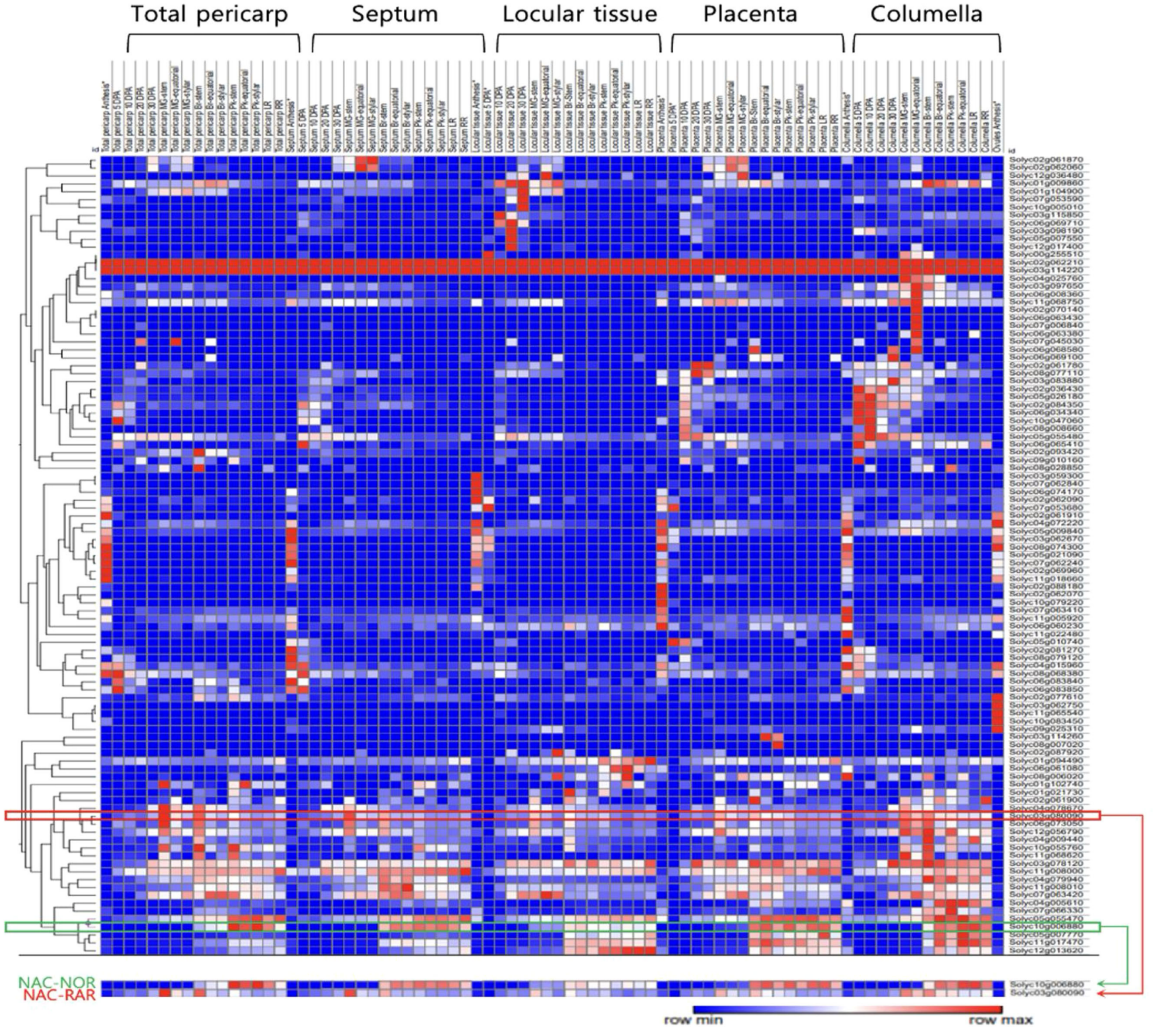
Comparative analysis of *RAR* and NAC family gene expression. Heat map showing expression profiles of 101 tomato NAC genes (rows) across fruit tissues (columns: total pericarp, septum, locular tissue, placenta, columella) from anthesis to the RR stage. The expression data of all NAC transcription factor family members was visualized and analyzed using Morpheus.

The expression profiles of NAC TF family members during tomato fruit development and ripening were visualized and analyzed using Morpheus (Broad Institute, https://software.broadinstitute.org/morpheus) based on absolute expression values (**Figure 5**). The heatmap displays temporal and spatial expression patterns across different fruit tissues, total pericarp, septum, locular tissue, placenta, and columella, using a red-to-blue gradient representing high to low expression levels. Among the NAC family members, the gene *RAR* exhibited a distinct expression profile compared to *NOR*. *NOR* showed minimal expression during early fruit development and was markedly upregulated from the BR stage onward, with transcript levels continuing to increase throughout the subsequent ripening phases. In contrast, *RAR* exhibited high transcript accumulation in the mature green stage, followed by a progressive decline in expression as ripening progressed.

### 3.2 Silencing of *RAR* accelerates plant senescence and climacteric ripening

To investigate the functional role of *RAR* in tomato fruit ripening, we generated transgenic RNA interference (RNAi) in which the expression of *RAR* was suppressed. Two independent T_0_ transgenic lines were obtained and validated by expression analysis (**Supplementary Figures 2A, B**). We selected homozygous line (RNAi-RAR 17-8) in T_2_ generation for further analysis. Quantitative real-time PCR analysis confirmed that transcript levels of *RAR* in MG stage fruits were significantly reduced in RNAi-RAR 17–8 compared to the WT, indicating effective gene silencing (**Supplementary Figure 2C**). Ripening-associated phenotypes were evaluated by monitoring fruit coloration at defined developmental stages. Under standard light intensity (82 µmol photons m^-^² s^-^¹), RNAi-RAR 17–8 fruits reached the BR stage at 28 days after pollination (DAP), approximately two days earlier than the azygous (AZ) control, which reached the BR stage at 30 DAP. Notably, under highlight intensity (235 µmol photons m^-^² s^-^¹), RNAi-RAR 17–8 fruits exhibited a markedly reduced time to reach the BR stage, accelerated overall ripening period in comparison to the AZ controls. The AZ lines reach the BR stage at 28 DAP, while RNAi-RAR 17–8 fruits reach the BR stage at 21 DAP, which is approximately 7 days earlier on average. The results demonstrated that RNAi-RAR 17–8 fruits exhibited accelerated ripening not only under standard light conditions but also showed a more pronounced reduction in the time required for fruit maturation under high light intensity (**Figure 1A**). Conversely, overexpression of *RAR* delayed fruit ripening. Transgenic RAR-OE fruits exhibited delayed pigmentation onset comparted to WT-J, reaching the BR stage at approximately 25 DAP, whereas WT-J fruits began ripening around 23 DAP under the same high light condition (235 µmol photons m^-^² s^-^¹), indicating that elevated *RAR* expression is sufficient to suppress the timing of climacteric ripening (**Figure 1B**).

To further evaluate the phenotypic consequences of *RAR* silencing, we assessed the overall vegetative growth status, including plant height and leaf senescence. Under standard light conditions (82 µmol photons m^-^² s^-^¹), RNAi-RAR 17–8 plants exhibited significantly increased height compared to AZ control plants. In contrast, under high light intensity (235 µmol photons m^-^² s^-^¹), the RNAi-RAR 17–8 plants showed reduced height relative to WT-J and AZ controls, accompanied by a more compact canopy structure (**Figures 1C, D**). Notably, RNAi-RAR 17–8 plants exhibited pronounced leaf yellowing under high light conditions, indicative of accelerated senescence. Consistently, chlorophyll content measured in the leaves of RNAi-RAR 17–8 plants was significantly lower than that of WT-J plants following equivalent durations of high light exposure (**Figure 1E**). To investigate whether *RAR* expression is regulated by light conditions and to evaluate transcriptional changes in ripening-related genes, we performed quantitative real-time PCR on MG stage fruits of WT-J grown under different light conditions. *RAR* expression was significantly reduced under high light, while *RIN* and several ethylene biosynthetic genes (*ACS2*, *ACS4*, *ACO1*) were significantly upregulated. *NOR* and *ACO3* showed increasing trends but without statistical significance (**Figure 1F**).

### 3.3 *RAR* accelerates fruit ripening through ethylene biosynthetic regulation

To investigate whether *RAR* regulates ethylene biosynthesis during fruit ripening, we measured ethylene production in WT-J and RNAi-RAR 17–8 tomato fruits by SGC (Sensor Gas Chromatograph) SGEA-P3-C1/C2/C3 (NISSHA, Osaka, Japan) (**Figure 2A**). Further analyses were conducted using samples from plants cultivated under high light conditions, which enhanced the phenotypic contrast between genotypes. RNAi-RAR 17–8 fruits exhibited a significant increase in ethylene production during the early stages of ripening (DAP 21–27), compared with WT-J fruits. Ethylene levels peaked around the DAP 33 in both genotypes and gradually declined thereafter. To further investigate the molecular basis of the observed phenotype, the expression levels of ethylene biosynthesis-related genes and ripening-associated transcription factors were quantified (**Figure 2B**). The transcripts of *ACS2*, *ACS4*, and *ACO1*, as well as the ripening regulators *NOR* and *RIN*, were significantly upregulated in RNAi-RAR 17–8 fruits compared to WT-J between DAP 23 and DAP 29.

### 3.4 Reciprocal regulation of *RAR* with ripening regulators *NOR* and *RIN*

Quantitative real-time PCR (qRT-PCR) was conducted to examine the transcriptional profiles of *RAR*, *NOR*, and *RIN*, across different organs, including root, stem, leaf, flower, and fruit at different developmental stages: immature green (IMG), MG, BR, and red ripe (RR) (**Figure 3A**). The results showed that *NOR*, and *RIN* were predominantly expressed in fruit tissues. However, *RAR* also exhibited detectable expression in vegetative organs, consistent with its previously observed effects on vegetative phenotypes (**Figure 1**). In fruits, *RAR* exhibited peak expression at the MG stage, followed by a decline at the BR stage. In contrast, *NOR* and *RIN* expression were markedly induced at the BR stage and remained high expression level at the RR stage.

To assess the ethylene responsiveness of *RAR* gene expression, WT-J fruits at the MG stage were treated with exogenous ethylene (1000 ppm, 24 h). The ethylene-inducible gene *ACS2* was used as a positive control. Ethylene treatment significantly upregulated *ACS2* expression, confirming the effectiveness of the treatment. Under the same conditions, expression of *RAR* was significantly reduced following ethylene exposure (**Figure 3B**).

To further assess the regulatory relationship between *RAR* and the known ripening-associated TFs *NOR* and *RIN*, the expression levels of *RAR* were analyzed in the *nor* and *rin* mutant backgrounds. In the *nor* mutant, *RAR* expression was comparable to WT-B at the MG stage but significantly increased at the BR stage (**Figure 3C**). In the *rin* mutant, *RAR* expression was significantly elevated at both MG (1.66 times higher than WT-B) and BR stages (5.92 times higher than WT-B) relative to wild-type (**Figure 3D**). These results indicate that *RAR* expression is negatively regulated by *NOR* and *RIN*, with a stronger effect observed in the *rin* mutant.

### 3.5 RAR interacts with NAC family TF and inactivates the promoters of ripening-related target genes

To investigate the interaction of RAR with other NAC family TF members, we performed BiFC in tobacco to further investigate the possible in planta interaction of RAR with NAC TF. The reconstitution of yellow fluorescence demonstrated the direct interaction of RAR-NOR in nucleus (**Figure 4A**), which also aligns with the characteristics of RAR and NOR as TFs.

To further elucidate the transactivation of RAR for ethylene biosynthesis related genes, a dual-luciferase reporter assay was performed by tobacco leaves to analyze the transcriptional activity of RAR with the promoters of *ACS2* and *ACO1*. The results showed that the relative LUC/REN ratio in tobacco leaves co-transformed with CaMV35S-*RAR* and CaMV35S-REN/*ACS2*-LUC showed significant inhibition of *ACS2* transcription compared with empty vectors (p<0.01), while RAR have no effect on the *ACO1* promoter (**Figure 4B**, **Supplementary Figure 4**). Given that the results above indicate a direct interaction between NOR and RAR proteins, we also assessed the transcriptional activity of the resulting heterodimer. When CaMV35S-*NOR* was co-expressed with CaMV35S-*RAR*, the activation effect of *ACS2* promoters was inhibited compared with the NOR protein occurring alone but remained significantly higher than the negative control (**Figure 4B**).

## 4 Discussion

### 4.1 *RAR* bridges fruit development and ripening by temporarily repressing autocatalytic ethylene production

Our study identifies *RAR*, a previously uncharacterized NAC TF, participates in fine-tuning the initiation of climacteric fruit ripening. *RAR* appears to integrate both developmental and environmental signals during the transition from fruit development to ripening (**Figure 1**). Previous studies have identified a number of ripening-related mutants, such as *nr*, *cnr*, *nor*, and *rin* (Lanahan et al., 1994; Manning et al., 2006; Gao et al., 2020; Vrebalov et al., 2002), which predominantly influence the BR stage by abolishing the climacteric ethylene burst, thus resulting in delayed ripening. However, few studies have addressed the regulatory mechanisms at the critical phase that before fruit transit to ripening. In this study, *RAR* was found to be highly expressed shortly before the BR stage (**Figure 3A**), precisely during the transition between development and ripening, helping to fill a key gap in our current understanding. Interestingly, *RAR* expression declines as ripening progresses in wild-type, in contrast to classical ripening-associated TFs such as *NOR* and *RIN*, which are upregulated at the BR stage (**Figure 3A**). In addition, the expression of *RAR* was decreased by exogenous ethylene treatment (**Figure 3B**), suggesting that the decreased expression of *RAR* at BR stage in wild-type is negatively regulated by autocatalytic ethylene burst.

Silencing of *RAR* led to a precocious onset of climacteric ripening, accompanied by a significant increase in ethylene production prior to the BR stage, while the ethylene production peak at the BR stage itself remained unaffected (**Figure 2A**). Furthermore, silencing of *RAR* resulted in the upregulation of key ethylene biosynthesis genes, including *ACS2*, *ACS4* and *ACO1*, at transition stages of fruit ripening (**Figure 2B**). These findings suggest that *RAR* functions as a repressor of ethylene biosynthesis during the transition into ripening (MG stage), and this repression can be released by a large increase in ethylene after the initiation of the ripening progress. To investigate the functional link between *RAR* and ethylene biosynthesis, a dual-luciferase assay was performed in tobacco leaves. Because RAR can physically interact with NOR (**Figure 4A**), we hypothesized that the two proteins may cooperatively regulate *NOR* target genes. Given that NOR has been shown to bind to the *ACS2* promoter (Gao et al., 2020), this assay was used to test whether RAR also affects *ACS2* transcriptional activity. The assay demonstrated that RAR significantly repressed the activity of the *ACS2* promoter, a key component of the ethylene biosynthesis pathway (**Figure 4B**). This repressive effect is consistent with transcriptomic data showing that *ACS2* expression is upregulated in *RAR* knockdown lines, supporting the notion that *RAR* acts as a negative regulator of ethylene biosynthesis during the early stage of fruit ripening. *RAR* did not repress *ACO1* promoter activity in the dual-luciferase assay (**Supplementary Figure 4**), implying that *ACO1* may be indirectly regulated via secondary factors downstream of *ACS2*-mediated ethylene signaling or other parallel pathways. Considering the extremely complex regulatory network governing fruit ripening, future studies will be needed to clarify how *RAR* is integrated into this multilayered transcriptional and hormonal framework. Taken together, our results provide evidence that a regulatory mechanism exists at the fruit transition stage to suppress autocatalytic ethylene production, which is essential for the coordinated initiation of ripening. From an ecological perspective, climacteric fruits produce large amounts of ethylene, which promotes ripening and can also induce fruit abscission and dispersal (Fukano and Tachiki, 2021). We hypothesize that such repression may serve as a developmental safeguard, allowing sufficient time for proper seed maturation before fruit abscission and dispersal. Such a mechanism may prevent the premature dispersal of physiologically immature seeds, which would otherwise be unable to establish the next generation.

From an applied perspective, *RAR* knockdown offers promising agronomic advantages. Our results demonstrate that silencing *RAR* accelerates the onset of fruit ripening without affecting final fruit size (**Figure 1A**), and its expression is confined to a short period just before the BR stage. This temporal specificity could minimize unintended impacts on early fruit development, allowing for earlier maturation without compromising yield potential. Minimizing the drawbacks remains a key challenge in development of commercially viable early-maturing varieties. In general, accelerated developmental programs may lead to desynchronization between the plant’s reproductive processes and the availability of key environmental resources such as light, temperature, water, and nutrients (McLain and Shure, 1990; Coen and Przemyslaw, 2024). This mismatch could result in suboptimal support for flowering and fruit development, potentially causing fruit drop, incomplete seed development, or other physiological disorders (Miller-Rushing et al., 2010). As pointed out in breeding literature, early-maturing cultivars are often associated with reduced yields, lower fruit quality, and compromised disease tolerance (Pan et al., 2023; Gradziel and Marchand, 2019). In our study, we observed that pericarp development was unaffected; however, *RAR* silencing disrupted normal seed development in a subset of fruits (**Supplementary Figure 3**). This indicates a degree of developmental uncoupling between seed and flesh tissues, which could be advantageous for the production of seedless or low-seed tomatoes, traits associated with improved texture and greater consumer preference. Because *RAR* is expressed at relatively high level during late fruit development and ripening initiation, it represents a strategic molecular target for shifting ripening timing while preserving essential fruit developmental processes. Further studies incorporating *RAR* knockout alleles into commercial tomato cultivars would allow for a more accurate assessment of *RAR*’s potential to accelerate fruit ripening without compromising yield or fruit quality.

### 4.2 *RAR* as a potential convergence point linking light intensity and hormonal regulation in tomato fruit ripening

Our study revealed that *RAR* could also play a role in modulating light-responsive developmental alterations. High light, a common abiotic factor in greenhouse and field conditions, is known to disrupt hormonal balance and alter the expression of ripening regulators including ethylene pathway genes. In this study, ethylene biosynthesis is frequently enhanced under high light conditions (**Figure 1F**). This observation is consistent with previous reports, which also demonstrated the induction of *ACS2*, *ACS4*, *ACO1* and *ACO4* (Zhang et al., 2020; Steelheart et al., 2022). Auxin modulates the balance with ethylene in light signaling pathways, coordinating hormone responses and carotenoid metabolism during tomato fruit ripening (Cruz et al, 2018). ABA levels often increase in response to elevated light, contributing to stomal regulation and abiotic stress acclimation (Lichtenthaler et al., 2013). Jasmonic acid (JA) accumulation is also promoted, enhancing defense-related gene expression (Ballaré, 2014). In contrast, gibberellin (GA) activity tends to decline under intense light, thereby limiting cell elongation and growth (Achard et al., 2007). Additionally, high light can suppress cytokinin (CK) signaling, reducing mitotic activity and meristem maintenance (Cortleven and Schmülling, 2015). These findings suggest that hormonal crosstalk under high light conditions plays a critical role in coordinating the timing and progression of fruit ripening, in which *RAR* contributes as a fine-tuning regulator within this complex hormonal network.

**Figure 6:**
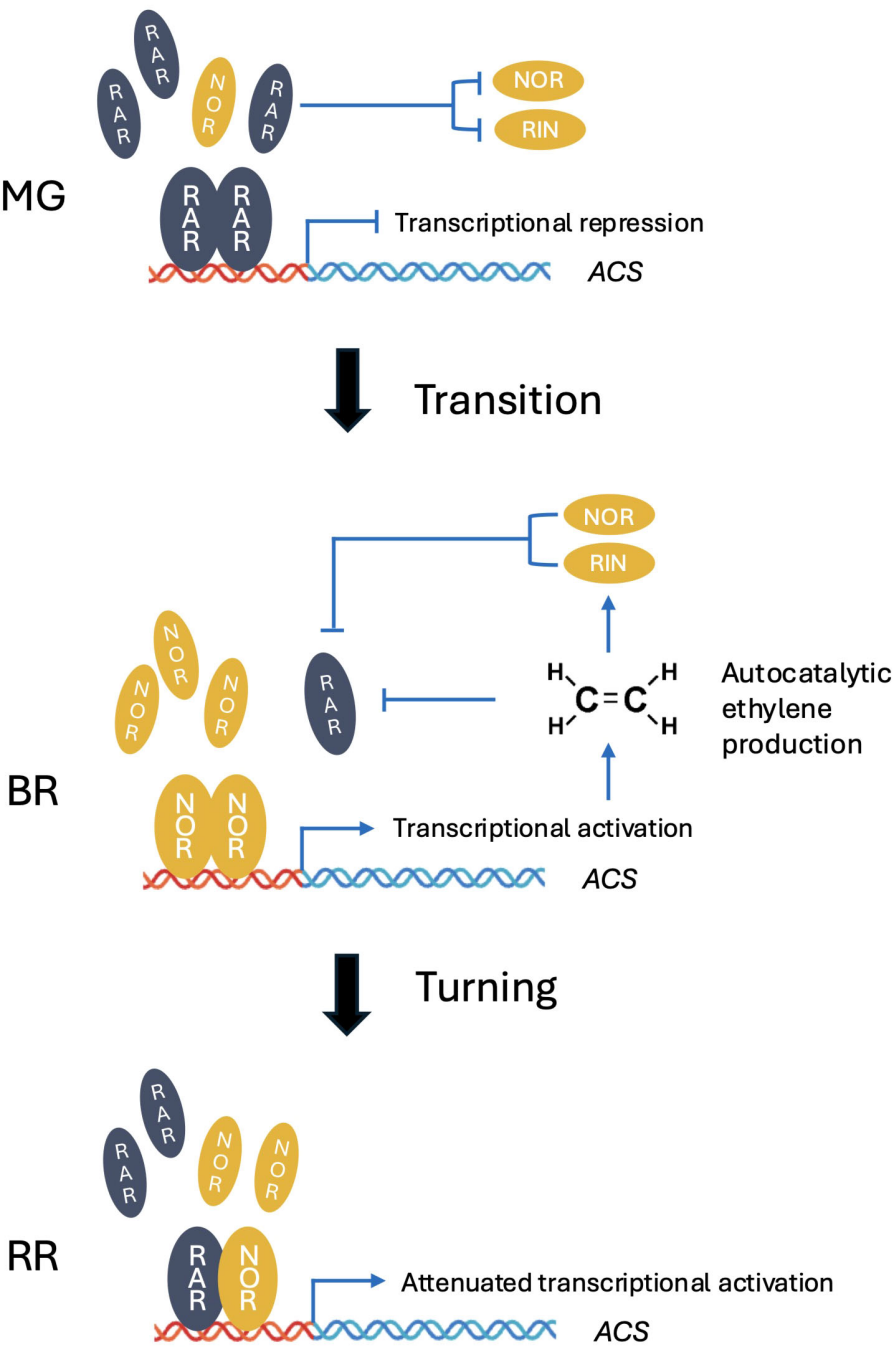
At the MG stage, high RAR abundance allows it to form homodimers, thereby repressing *ACS2* transcription and preventing premature ethylene biosynthesis. Once the autocatalytic ethylene synthesis loop is initiated, ethylene, together with NOR and RIN, represses RAR expression, thus relieving this inhibition and enabling full activation of the ripening regulatory cascade. At the RR stage, RAR levels remain lower but still sufficient, and may tend to form heterodimers with NOR, thereby attenuating the transcriptional activation of *ACS2* and fine-tuning ethylene production during late ripening.

In our study, *RAR* transcript levels were markedly higher in vegetative tissues such as stems, leaves, and flowers compared with fruits (**Figure 3A**). This suggests that the primary function of *RAR* may extend beyond fruit ripening to broader roles in plant growth and development. Consistently, RNAi-RAR plants exhibited altered vegetative traits, including changes in plant height and reduced chlorophyll content under high light (**Figures 1C–E**), indicating that *RAR* contributes to light-mediated regulation of photosynthetic capacity and vegetative growth. Expression analysis in MG stage WT-J fruits showed that high light did not significantly affect *NOR* and *ACO3* transcript levels (**Figure 1F**), although the observed trends are consistent with previous reports that *NOR* activates *ACO3* transcription (Wang et al., 2018). By contrast, under high light, *RIN*, *ACS2*, *ACS4*, and *ACO1* were significantly upregulated. These results suggest that high light promotes ethylene biosynthetic gene expression in developing fruits, coinciding with reduced expression of the negative regulator *RAR*. Consistently, in *RAR*-silenced fruits, *RIN*, *ACS2*, *ACS4*, and *ACO1* were also upregulated (**Figure 2B**), providing complementary evidence. Thus, the combined effects of high light and *RAR* silencing likely explain the accelerated growth cycle and faster fruit ripening observed under these conditions (**Figure 1**). This pattern is consistent with our competitive dimerization model (**Figure 6**), in which high light reduces RAR levels, thereby shifting the dimer composition toward NOR–NOR homodimers that exhibit stronger transcriptional activation capacity than RAR–NOR heterodimers. In this scenario, the repressive influence of RAR is attenuated, allowing the full activation potential of NOR to be realized and thereby enhancing the induction of downstream ethylene pathway genes without requiring an increase in NOR transcript levels.

The dual role of *RAR* in both ethylene-mediated ripening and light sensitivity suggests the gene function may act as a convergence point linking environmental signals with fruit maturation processes. Further investigation is required to elucidate the molecular mechanisms by which *RAR* modulates these processes, but our results provide preliminary evidence that *RAR* may help fine-tune the timing of ripening in response to changing environmental conditions, thereby maintaining reproductive success and crop quality under stress-prone scenarios. The responsiveness of *RAR* to light conditions suggests its potential utility in low-latitude open-field cultivation or controlled-environment agriculture, such as plant factories, where intense or artificial lighting is common. Accelerating the time to harvest under such conditions could contribute to increased cropping cycles and production efficiency.

### 4.3 Interregulation of *RAR*, *NOR* and *RIN* during the transition to ripening

The transcriptional regulation governing the transition from fruit development to ripening, often referred to as the transition stage, remains poorly defined. The induction of autocatalytic ethylene biosynthesis is known to involve a complex regulatory cascade, including mutual regulation and feedback between key TFs such as *NOR* and *RIN* (Fujisawa et al., 2013; Giovannoni et al., 2017; Liu et al., 2024). While *NOR* and *RIN* are well established as master regulators activated at the BR stage, our findings show that *RAR* participates in this intricate regulatory framework, serving as a molecular gatekeeper to delay ripening onset. In RNAi-RAR fruits, we observed precocious activation of *NOR* and *RIN* expression, which was accompanied by increased ethylene production prior to the BR stage (**Figure 2**). These results suggest that *RAR*-mediated repression of *NOR* and *RIN* expression helps to prevent untimely entry into the ripening phase in wild-type. On the other hand, the expression of *RAR* is elevated at both the MG and BR stages in the *rin* mutant, and also at the BR stage in the *nor* mutant (**Figure 3**). These expression patterns suggest the presence of a dual-layered negative feedback mechanism in which both ethylene and its upstream transcriptional activators (*NOR* and *RIN*) contribute to the suppression of *RAR* expression. This suppression likely facilitates the transition from a repressive to an active ripening state (**Figure 6**).

NAC TFs typically function as homodimers or heterodimers, and dimerization is essential for stable DNA binding (Olsen et al., 2005). Supporting this, BiFC assays confirmed that RAR physically interacts with NOR in the nucleus, forming a heterodimer (**Figure 4A**). Dual-luciferase reporter assays further showed that co-expression of *RAR* with *NOR* significantly reduced *NOR*-mediated activation of the *ACS2* promoter compared to *NOR* alone (**Figure 4B**). This evidence indicates that RAR may suppress NOR activity by preventing NOR homodimer formation, thereby attenuating its transcriptional activation potential. We also observed a temporal shift in gene expression around the BR stage. At the MG stage, the relatively high expression of *RAR* suggests that its repressive effect predominates, whereas at the BR and RR stages, the substantially elevated *NOR* expression drives ethylene biosynthesis and becomes the dominant factor (**Figure 3A**; **Supplementary Table 4**). This inverse relationship suggests a regulatory transition from repression to activation of ethylene biosynthetic genes. Notably, the downregulation of *RAR* coincides with the onset of autocatalytic ethylene production, implying that ethylene signaling may actively repress *RAR* expression to relieve its inhibitory effect on ripening. This interpretation is further supported by our finding that exogenous ethylene treatment significantly reduces *RAR* transcript levels (**Figure 3B**).

Taken together, our expression and functional analyses support a model in which *RAR* functions as a fine-tuning regulator during the early ripening stages. At the MG stage, high RAR abundance allows it to form homodimers, thereby repressing *ACS* transcription and preventing premature ethylene biosynthesis. Once the autocatalytic ethylene synthesis loop is initiated, ethylene, along with *NOR* and *RIN*, acts to repress *RAR* expression, thereby relieving this inhibition and allowing full activation of the ripening regulatory cascade. At RR stage, RAR levels remain lower but still sufficient, and may trend to form heterodimers with NOR, thereby attenuating the transcriptional activation of *ACS* (**Figure 6**). This stage-specific interaction reflects a dynamic balance between *NOR* and *RAR*, where the relative abundance of each factor determines the extent of heterodimer formation and thus modulates the strength of *ACS* activation during successive ripening stages. This study uncovers a previously uncharacterized temporal regulatory switch in fruit ripening, where in the dynamic balance between different NAC transcription factors modulates the timing and amplitude of ethylene biosynthetic gene expression. Such a mechanism underscores the complexity and precision of transcriptional control required for proper ripening progression.

## Data Availability

The original contributions presented in the study are included in the article/**Supplementary Material**, further inquiries can be directed to the corresponding author/s.

## Supplementary material

Supplementary Figure 1 Comparison of the maximum expression level NAC family genes during fruit development. The candidate gene Solyc03g080090, which is proposed to regulate fruit ripening, is the third most highly expressed gene within the NAC family. Expression levels were compared across developmental stages and tissue types (total pericarp, septum, locular tissue, placenta, columella) using the highest mean value observed in each.

Supplementary Figure 2 Expression analysis and ripening phenotypes of RAR transgenic tomato lines. (A) Fruit phenotype of WT-J, AZ (azygous) and RNAi-RAR-12, RNAi-RAR-17 T0 generation exposed to controlled light conditions of 82 µmol photons m^−2^ s^−1^, respectively. Bar = 1 cm. (B) Relative expression level of RAR in WT-J, RNAi-RAR-12 and RNAi-RAR-17 T0 generation MG fruits. Error bars represent the standard deviations. Significant differences were determined by two-tailed Student’s t test (**p < 0.01), n = 3. (C) Relative expression level of RAR in WT-J and RNAi-RAR 17-8 MG and BR fruits. Error bars represent the standard deviations. Significant differences were determined by two-tailed Student’s t test (**p < 0.01), n = 4. (D) Relative expression level of RAR in WT-J and RAR-OE 0-11 tomato leaf.

Supplementary Figure 3 RAR Silencing Impairs Seed Development. (A) Phenotype of seeds in RNAi-RAR 17-8 fruits at DAP 30. Bar=1cm. (B) Averaged mature seeds number per fruit in WT-J and RNAi-RAR 17-8 fruits at DAP 30. Significant differences were determined by two-tailed Student’s t test (**p < 0.01), n = 10.

Supplementary Figure 4 RAR have no effect on the transcriptional activity of ACO1. (A) Dual-luciferase assays showing the effect of RAR on the transcriptional activity of ACO1. RAR and NOR were driven by 35S promoter as an effector. 1kb of ACO1 promoter were used to drive LUC as a reporter. (B) The activation of ACO1 promoter by RAR was shown by the ratio of LUC to REN. Data are the mean ± SD of 5 biological replicates. Error bars of figure on the right represent the standard deviations. Letters indicate significant differences according to Tukey’s test (p < 0.05), n = 5.
