# Supplementary figures and images for: A novel NAC transcription factor mediates negative regulation of early ethylene production and ripening in tomato fruits
Source: Front Plant Sci. 2025 Nov 19;16:1696915. doi: 10.3389/fpls.2025.1696915 (PMC12672533; doi:10.3389/fpls.2025.1696915)

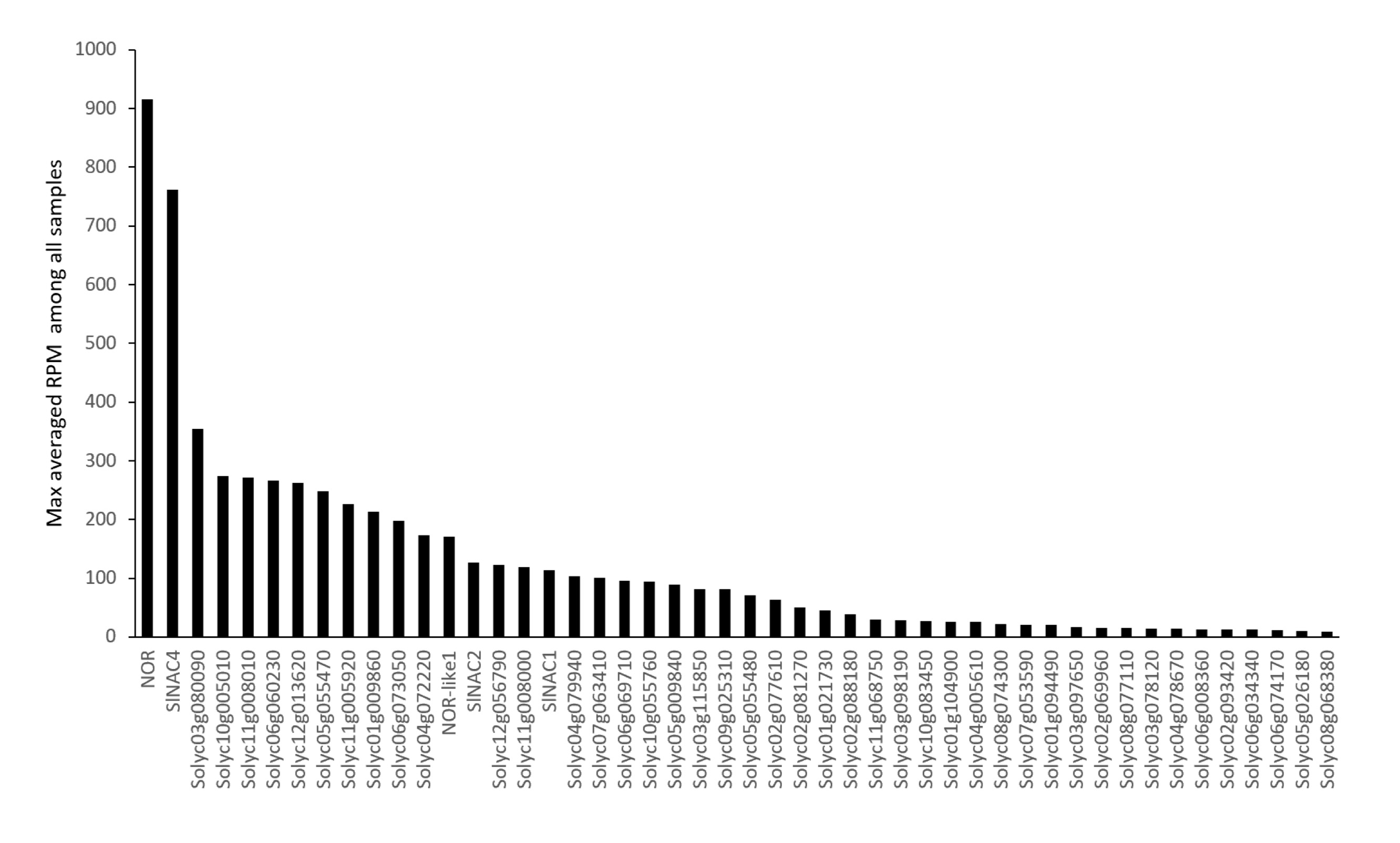

Supplement: Supplementary Figure 1 — Comparison of the maximum expression level NAC family genes during fruit development. The candidate gene Solyc03g080090, which is proposed to regulate fruit ripening, is the third most highly expressed gene within the NAC family. Expression levels were compared across developmental stages and tissue types (total pericarp, septum, locular tissue, placenta, columella) using the highest mean value observed in each. [file Image1.jpeg]

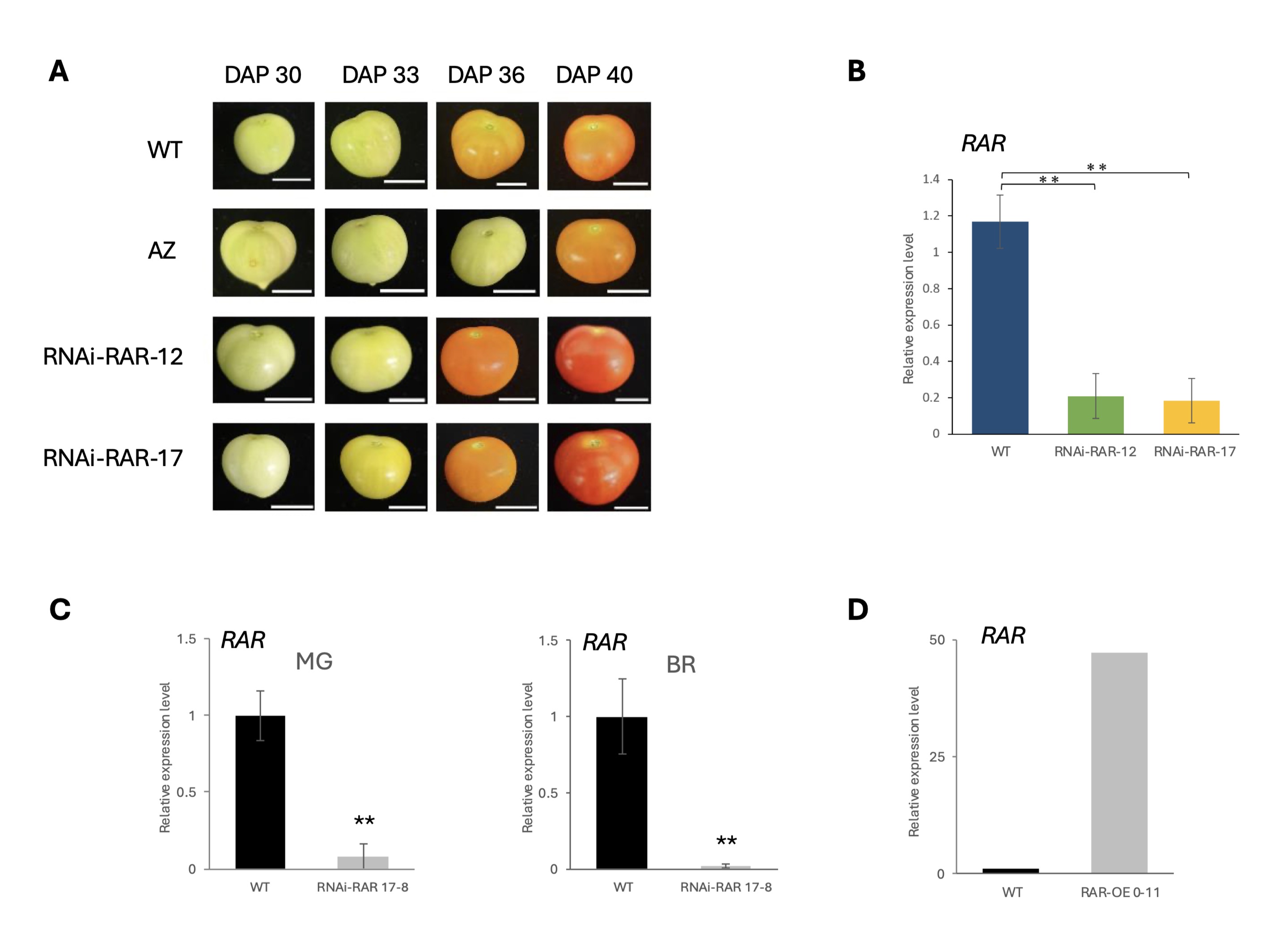

Supplement: Supplementary Figure 2 — Expression analysis and ripening phenotypes of RAR transgenic tomato lines. (A) Fruit phenotype of WT-J, AZ (azygous) and RNAi-RAR-12, RNAi-RAR-17 T0 generation exposed to controlled light conditions of 82 µmol photons m−2 s−1, respectively. Bar = 1 cm. (B) Relative expression level of RAR in WT-J, RNAi-RAR-12 and RNAi-RAR-17 T0 generation MG fruits. Error bars represent the standard deviations. Significant differences were determined by two-tailed Student’s t test (**p < 0.01), n = 3. (C) Relative expression level of RAR in WT-J and RNAi-RAR 17-8 MG and BR fruits. Error bars represent the standard deviations. Significant differences were determined by two-tailed Student’s t test (**p < 0.01), n = 4. (D) Relative expression level of RAR in WT-J and RAR-OE 0-11 tomato leaf. [file Image2.jpeg]

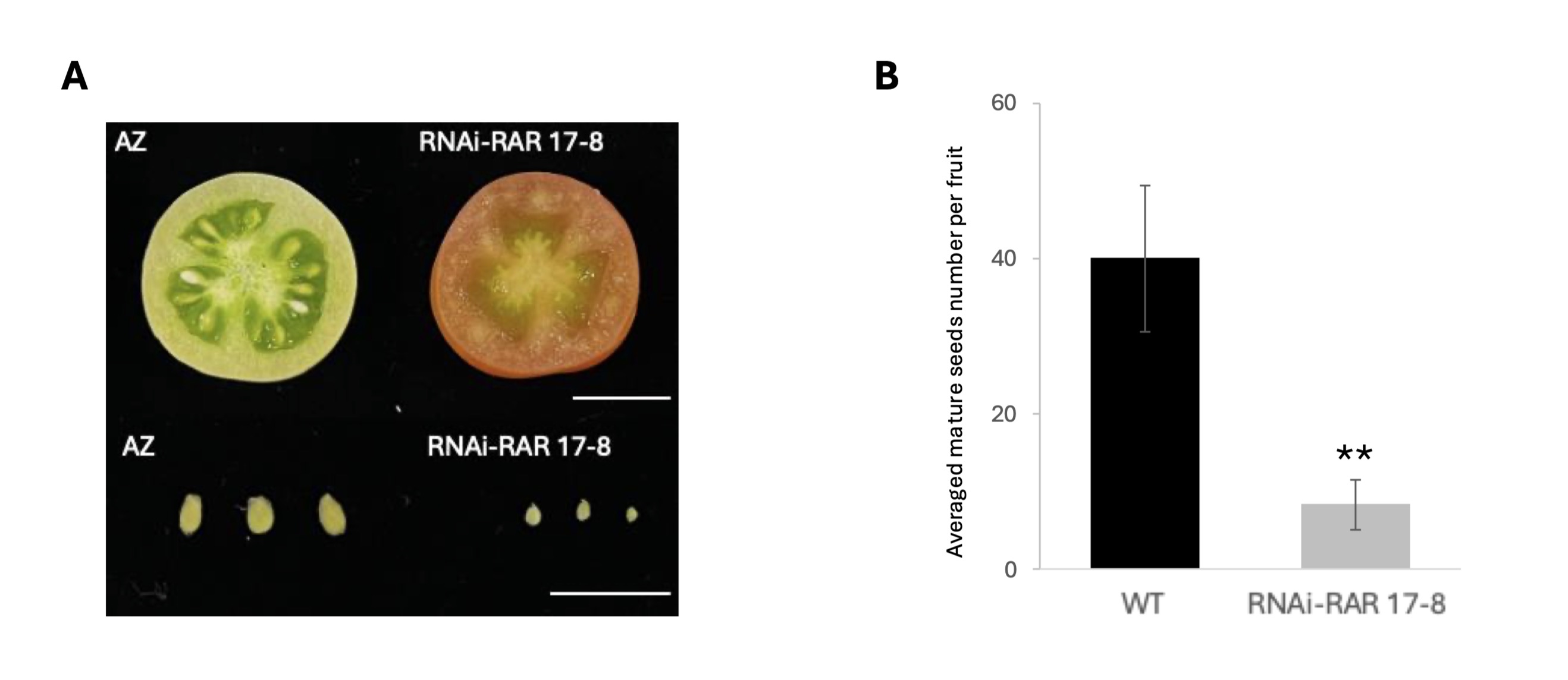

Supplement: Supplementary Figure 3 — RAR Silencing Impairs Seed Development. (A) Phenotype of seeds in RNAi-RAR 17-8 fruits at DAP 30. Bar=1cm. (B) Averaged mature seeds number per fruit in WT-J and RNAi-RAR 17-8 fruits at DAP 30. Significant differences were determined by two-tailed Student’s t test (**p < 0.01), n = 10. [file Image3.jpeg]

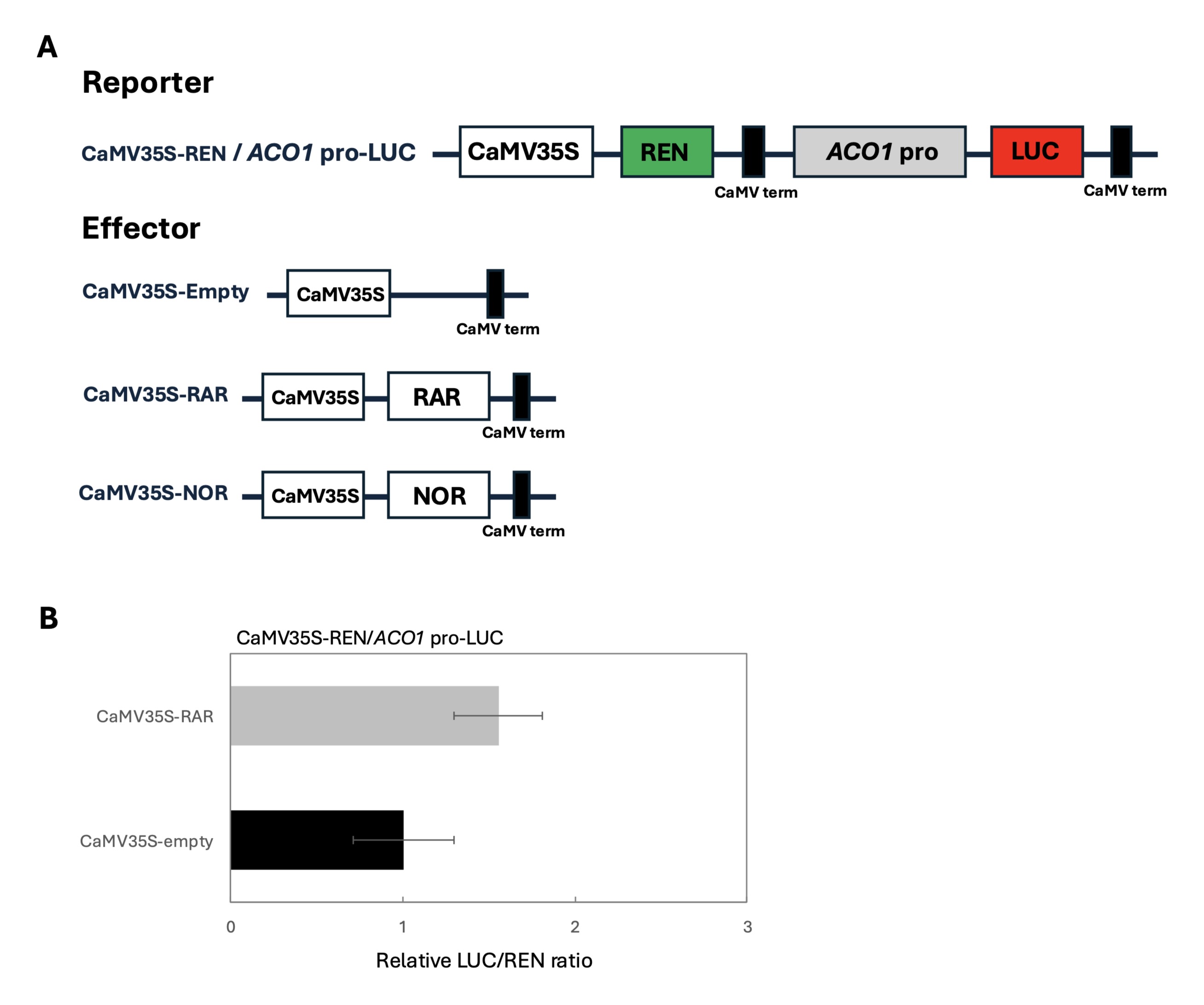

Supplement: Supplementary Figure 4 — RAR have no effect on the transcriptional activity of ACO1. (A) Dual-luciferase assays showing the effect of RAR on the transcriptional activity of ACO1. RAR and NOR were driven by 35S promoter as an effector. 1kb of ACO1 promoter were used to drive LUC as a reporter. (B) The activation of ACO1 promoter by RAR was shown by the ratio of LUC to REN. Data are the mean ± SD of 5 biological replicates. Error bars of figure on the right represent the standard deviations. Letters indicate significant differences according to Tukey’s test (p < 0.05), n = 5. [file Image4.jpeg]
