# Supplementary material for: A novel NAC transcription factor mediates negative regulation of early ethylene production and ripening in tomato fruits
Source: Front Plant Sci. 2025 Nov 19;16:1696915. doi: 10.3389/fpls.2025.1696915 (PMC12672533; doi:10.3389/fpls.2025.1696915)
Supplement: Supplementary file 5 [file Table1.docx]

**Supplementary**

**Table S1. Component of MS culture medium.**

|  | MS medium (1 L) | Agrobacterium infection solution (100 mL) | Co-culture medium (1 L) | Callus medium (1 L) | Shoot medium (1 L) | Root medium (1 L) |
| --- | --- | --- | --- | --- | --- | --- |
| MS stock | 4.4 g | 0.44 g | 4.4 g | 4.4 g | 4.4 g | 2.2 g |
| Sucrose (3 %) | 30 g | 3 g | 30 g | 30 g | 30 g | 15 g |
| Gelite (0.3 %) | 3 g |  | 3 g | 3 g | 3 g | 3 g |
| pH | 5.6-5.8 | 5.6-5.8 | 5.6-5.8 | 5.6-5.8 | 5.6-5.8 | 5.6-5.8 |
| Zeatin (10 mg/mL) |  |  | 150 μL | 150 μL | 100 μL |  |
| Kanamycin (100 mg/mL) |  |  |  | 1 mL | 1 mL | 0.5 mL |
| Augmentin (1 tablet / 375 mg) |  |  |  | 1 tablet | 1 tablet | 1 tablet |
| Acetosyringone (100 mM) |  |  | 100 μL | 100 μL |  |  |
| Mercaptoethanol (10 mM) |  |  | 100 μL |  |  |  |

**Table S2. Primers for vector construction.**

| Construct | Primer | Sequence (5' - 3') |
| --- | --- | --- |
| RNAi-*RAR* | Fw | ATTACGCCAAGCTTGCATGC |
|  | Re | CGGGGATCCTCTAGAGTCGA |
| pRI201-cEYFP-*RAR-*C1 | Fw | CGGAGGTGGTGTCGAATGGCCGTACTTCCTGGAG |
|  | Re | ATTATCATTAGTCGACTATACACAGAGTCTAAAGCAACTC |
| pRI201-nEYFP-*NOR*-C1 | Fw | GGACGGTGGTGTCGAATGGAAAGTACGGATTCATCAACC |
|  | Re | ATTATCATTAGTCGAAGAGTACCAATTCATGCCAGTAAC |
| NPTII | Fw | ATGATTGAACAAGATGGATTGCAC |
|  | Re | TCAGAAGAACTCGTCAAGAAGGCG |
| pGreen II 0800-LUC-*ACS2* | Fw | CTATAGGGCGAATTGGGTACCATTACCAGTAGTACCATTGTATTCTCAACT |
|  | Re | TGTTTTTGGCGTCTTCCATGGTGTGAGGGATATATATAAGGGGAAAG |
| pGreen II 0800-LUC-*ACO1* | Fw | CTATAGGGCGAATTGGGTACCGGTTTAGGATTCTATAAATAGAGGCATG |
|  | Re | TGTTTTTGGCGTCTTCCATGGAAATAATGAGGCTTTGAAAGGGTATTTA |
| pBI121-*RAR* | Fw | TCTAGAGGATCCCCATGGCCGTACTTCCTGG |
|  | Re | GATCGGGGAAATTCGCTATACACAGAGTCTAAAGCAAC |
| pBI121-*NOR* | Fw | CGGGAATTGCTACCGATGGAAAGTACGGATTCATCAACC |
|  | Re | CCGGGGATCCTCTAGTTAAGAGTACCAATTCATGCCAG |

**Table S3. Primers for qRT-PCR.**

| Target gene | Primer name | Sequence (5'-3') | Reference |
| --- | --- | --- | --- |
| *SlUbiquitin* | SlUbiquitin-F | CACCAAGCCAAAGAAGATCA | Takayama et al., 2015 |
|  | SlUbiquitin-R | TCAGCATTAGGGCACTCCTT |  |
| *ACS2* | ACS2-F | GGAGGTTCGTAGGTGTTGAG | Mantelin et al., 2013 |
|  | ACS2-R | TAATGGTGAGGGAGGAATAGGT |  |
| *ACS4* | ACS4-F | AACAAGCACAATGGAAGAGGA | Mantelin et al., 2013 |
|  | ACS4-R | CGCACTACGAGCAAGGAAT |  |
| *ACO1* | ACO1-F | ACCATGTCCTAAGCCCGATTT | Shinozaki et al., 2015 |
|  | ACO1-R | ATTCGTGTCCCGTCTGTTTGT |  |
| *ACO3* | ACO3-F | CATGTCCTAAGCCCGATTTGA | Shinozaki et al., 2015 |
|  | ACO3-R | CCGAGTCCCATCTGTTTGTG |  |
| *NOR* | NOR-F | TACCGGAACCGACAAGC | Chen et al., 2022 |
|  | NOR-R | ATATCATGCAAATCATCTATGGACC |  |
| *RIN* | RIN-F | CATGGCATTGTGGTGAGCAAAGTGT | Wang et al., 2018 |
|  | RIN-R | AGCATCATGTGTTGATGGTGCTGC |  |
| *RAR* | RAR-F | TGTTCGCAATAATGTAGCAAG | This study |
|  | RAR-R | CGACACTCAACTGGTCCGA |  |

**Table S4. Absolute expression levels (Reads Per Million, RPM) in the total pericarp of the equatorial region at different fruit developmental stages.**

| Gene ID | Gene name | MG | BR | PINK | RR |
| --- | --- | --- | --- | --- | --- |
| *Solyc10g006880* | *NOR* | 11.28 | 408.34 | 797.22 | 494.92 |
| *Solyc03g080090* | *RAR* | 205.79 | 157.13 | 136.87 | 148.42 |

Data were retrieved from the Tomato Expression Atlas (Fernandez-Pozo et al., 2017).
